# Efficacy and safety of doravirine/lamivudine/tenofovir disoproxil fumarate in HIV treatment: a real-world single-center study in China

**DOI:** 10.3389/fmed.2025.1575411

**Published:** 2025-05-29

**Authors:** Aixin Li, Letian Liu, Hongwei Zhang, Xi Wang, Zaicun Li, An Liu, Lili Dai, Jingji Zhang, Yue Gao, Jiangzhu Ye, Jianwei Li, Lijun Sun

**Affiliations:** ^1^Center for Infectious Diseases, Beijing Youan Hospital, Capital Medical University, Beijing, China; ^2^Chinese Association of STD and AIDS Prevention and Control, Care and Treatment Committee, Beijing, China

**Keywords:** HIV, doravirine, DOR/3TC/TDF, real-world, antiretroviral therapy

## Abstract

**Background:**

The single-tablet regimen Doravirine/Lamivudine/Tenofovir Disoproxil Fumarate (DOR/3TC/TDF) has been included in international guidelines and recommendations and was approved by China’s National Medical Products Administration (NMPA) in early 2021 for adult human immunodeficiency virus (HIV)-1 infections. This study presents real-world results of a retrospective analysis of patients who initiated DOR/3TC/TDF at a Chinese HIV center.

**Methods:**

This retrospective analysis was carried out on patients who received DOR/3TC/TDF (initial or switch) at the outpatient clinic of the Infection Center in Beijing Youan Hospital in China. Patients’ baseline characteristics, reasons for switching to DOR/3TC/TDF, along with the preliminary clinical, laboratory – based efficacy, safety, and tolerability data, were collected. All evaluations were in strict accordance with the protocols of our center. The statistical analysis was mainly descriptive, aiming to assess the changes in laboratory parameters from the baseline to the data – collection deadline, which was December 31, 2024.

**Result:**

From May 16 to October 29, 2024, 205 patients were prescribed DOR/3TC/TDF, either as an initiation or a switch. The cohort consisted mainly of males (96.1%), with a median age of 36.0 (31.0, 41.0) years. By the analysis deadline, the entire group had used DOR/3TC/TDF for 149.0 (90.0, 202.0) days. Among them, 40 patients were treatment-naïve, with a median HIV-1 ribonucleic acid (HIV-1 RNA) of 4.1 (3.7, 4.6) log_10_ copies/mL. At weeks 12 and 24, 64.5% [95% confidence interval (CI): 45.4, 80.8%] and 91.3% (95% CI: 72.0, 98.9%) of the participants achieved HIV-1 RNA < 50 copies/mL. Subgroup analysis showed that high viral load (VL) (≥10^5^ copies/mL) and low CD4 counts (< 200 cells/μL) at baseline did not affect virological efficacy. The results of immune reconstitution were also satisfactory, with CD4 counts increased from 350 (264, 465) cells/μL at baseline to 541.0 (415.8, 789.5) cells/μL by the end of the follow-up (*p* > 0.05). 165 patients (80.5%) had treatment experience, and the most common cause for switching was treatment simplification (40%). After the switch, an equally high proportion of patients [97.6% (95% CI: 93.7, 99.3%) vs. 96.4% (95% CI: 92.2, 98.7%)] achieved HIV-1 RNA undetectable or <50 copies/mL (*p* > 0.05). Compared to baseline, there were no significant changes in liver enzymes and renal function (*p* > 0.05), while body weight, random blood glucose and blood lipid levels decreased significantly (*p* < 0.05). Among patients with central nervous system (CNS) symptom, both the Pittsburgh Sleep Quality Index (PSQI) and the Hospital Anxiety and Depression Scale (HADS) scores, as well as the proportion of patients with scores greater than 7 points, decreased significantly post-switch (*p* < 0.05).

**Conclusion:**

We provided an observational report on the effectiveness and safety of the short-term use of DOR/3TC/TDF in routine clinical practice.

## Introduction

Integrase inhibitors (INSTIs) had been recommended by major antiretroviral therapy (ART) guidelines, including the United States Department of Health and Human Services (DHHS), European AIDS Clinical Society (EACS) and Chinese Guideline for Diagnosis and Treatment of HIV/AIDS (2024 Edition), as first-line agents for HIV-1 (1–3). Significant weight gain was consistently observed with dolutegravir (DTG), bictegravir (BIC) and elvitegravir, with an average weight gain of about 4 kg by 96 weeks, exceeding non-nucleoside reverse transcriptase inhibitor (NNRTI) or protease inhibitor (PI) -based regimens (4). Weight gain might have represented a therapeutic effect among individuals with advanced immunosuppression, however, it increases the risk of overweight, obesity and cardiovascular disease (5, 6). Clinical data showed that doravirine (DOR) had minimal impact on lipids, and it could have been a better regimen for subjects with metabolic issues (7).

DOR was a novel third generation NNRTI and was first approved by the United States Food and Drug Administration (FDA) and the European Medicines Agency (EMA) in 2018, and then approved by the China National Medical Products Administration (NMPA) in early 2021. It was launched in China for the treatment of HIV-1 infections in adults without resistance to NNRTIs or lamivudine (3TC) or tenofovir disoproxil fumarate (TDF). Both single-dose DOR and fixed-dose DOR/3TC/TDF were recommended by international and Chinese guidelines and recommendations (1–3).

This retrospective analysis described the baseline characteristics and evaluated the efficacy and tolerability of DOR/3TC/TDF in HIV-1 infected individuals at a Chinese clinical center.

## Methods

### Ethics statement

This study was approved by the Ethics Committee of Beijing Youan Hospital, Capital Medical University (No. 2022-086) and complied with the Helsinki Declaration (revised in 2013). As a retrospective study, informed consent was not required.

### Study design and patients

This retrospective analysis study included HIV-1 infected individuals followed up at the Infection Center of Beijing Youan Hospital, Capital Medical University. We selected patients who received DOR/3TC/TDF (either as an initial treatment or a switch) between May 16 and October 29, 2024. Patients with incomplete data, prior or current resistance to NNRTIs, or pregnancy were excluded. All clinical evaluations were performed in accordance with the follow-up requirements of our treatment center, without additional testing or examination. Data were collected by consulting electronic medical records by December 31, 2024.

### Data collection

Participants who initiated/switched to DOR/3TC/TDF regimens after enrollment were evaluated. Demographics (age, sex, weight), clinical data (ART initiation time, previous ART regimen, reasons for protocol adjustment), transmission route, coinfection, comorbidities, and laboratory test results (complete blood count, liver function, kidney function, random blood glucose, lipid profile, CD4 counts, and HIV-1 RNA) were obtained from medical records.

For patients who adjusted their ART regimen due to central nervous system (CNS) symptoms, we collected sleep disorder, anxiety and depression symptom scores by using the Pittsburgh Sleep Quality Index (PSQI) scores and the Hospital Anxiety and Depression Scale (HADS) scores. The PSQI was used to assess sleep quality. The HADS included two subscales, the HADS-A and HADS-D, which were intended to measure mutually exclusive levels of anxiety and depression, respectively. A total score of >7 on either indicated varying degrees of severity.

### Statistical analysis

Continuous variables with a normal distribution were analyzed with the *t*-tests and reported as the mean ± standard deviation (SD). Continuous variables with abnormal distribution were analyzed using the Mann–Whitney U-test and described as the median and interquartile range (IQR). Virological suppression rates and their variability were estimated using two-sided exact Clopper-Pearson 95% confidence intervals (CIs) based on the binomial distribution. Given the small sample sizes in subgroup analyses (e.g., baseline HIV-1 RNA ≥ 10^5^ copies/mL: *n* = 4 at Week 12), the 95% CIs were intentionally wide to reflect uncertainty (e.g., 2/4 [50.0, 95% CI: 6.7, 93.3%] at Week 12). For larger subgroups (e.g., baseline HIV-1 RNA < 10^5^ copies/mL: *n* = 27 at Week 12), the 95% CI widths narrowed (e.g., 18/27 [66.7, 95% CI: 45.7, 83.6%]). Subgroup comparisons were evaluated using two-sided Fisher’s exact tests, with no adjustments for multiple comparisons. All statistical tests and CIs were nominal and interpreted descriptively due to the exploratory nature of subgroup analyses. Statistical analyses were performed using SPSS 26.0 (IBM Corp., Armonk, NY, United States), GraphPad Prism 9.5.0 (GraphPad Software, San Diego, CA, United States), and R 4.2.3 (8). A *p* value < 0.05 (two tailed) was considered statistically significant.

## Results

**Figure 1:**
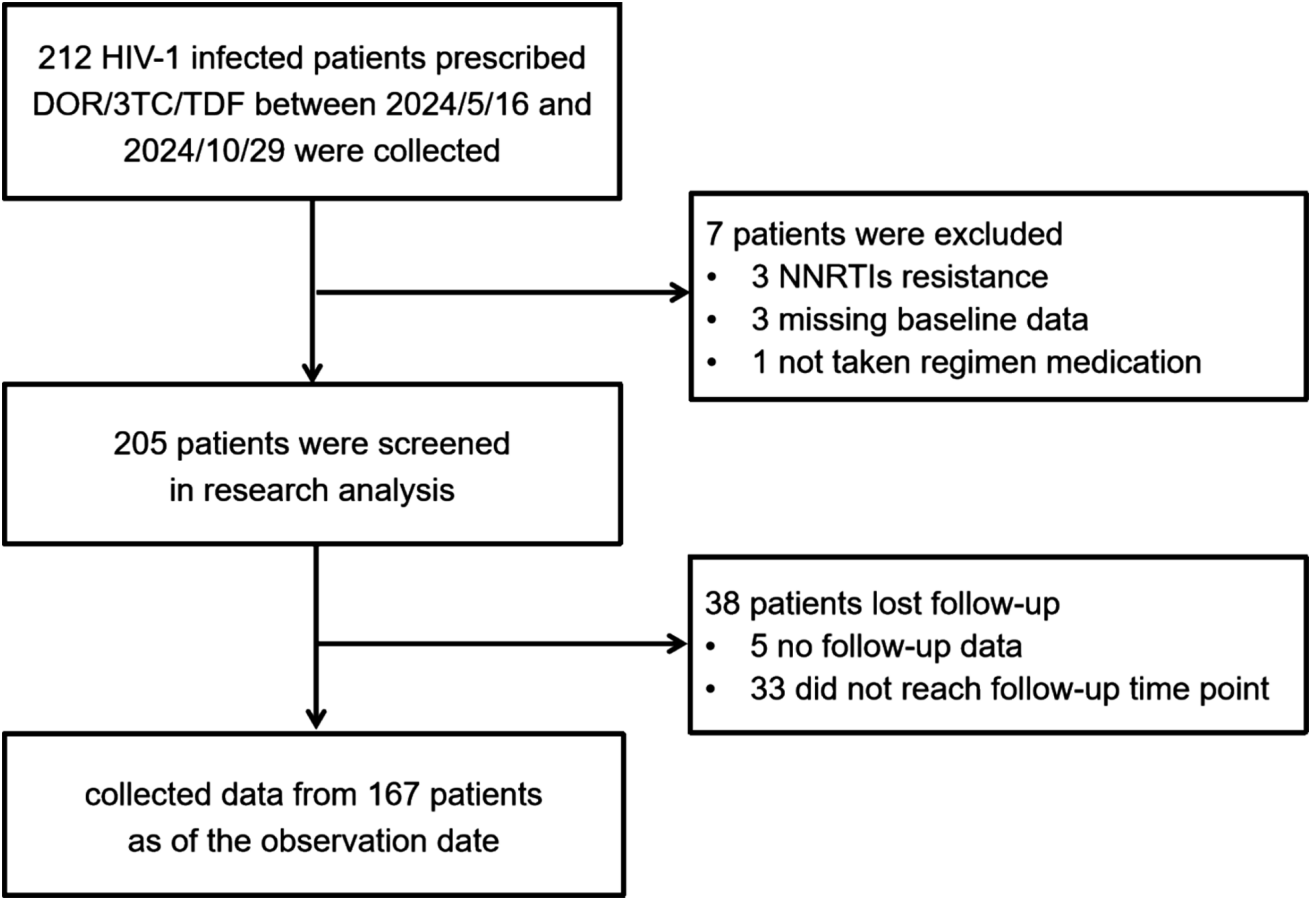
The flow chart of recruiting patients.

Between May 16 and October 29, 2024, 212 patients received outpatient treatment at Beijing Youan Hospital, were prescribed DOR/3TC/TDF. Seven patients were excluded: three had NNRTIs resistance, three lacked baseline data, and one who did not take the medication. Thus, 205 patients were included in the study analysis (Table 1). During the follow-up, 5 patients were lost to followed-up after the switch, and 33 did not reach the required follow-up time. Complete follow-up data were collected for 167 patients (Figure 1).

**Table 1:** Baseline characteristics of total patients.

Variables	Overall (*N* = 205)	Treatment experienced (*N* = 165)	Treatment naïve (*N* = 40)
Demographics			
Age, years, median (IQR)	36.0 (31.0, 41.0)	36.0 (32.0, 41.0)	33.5 (29.8, 41.2)
Age≥50 years, *n* (%)	26 (12.7)	20 (12.1)	6 (15.0)
Male, *n* (%)	197 (96.1)	159 (96.4)	38 (95.0)
Weight, kg, median (IQR)	70.0 (63.5, 80.0)	70.0 (65.0, 80.0)	70.0 (60.0, 76.5)
Co-infection, *n* (%)			
HBV	5 (2.4)	2 (1.2)	3 (7.5)
HCV	1 (0.5)	1 (0.6)	0 (0.0)
Syphilis	20 (9.8)	8 (4.8)	12 (30.0)
Genital warts	3 (1.5)	1 (0.6)	2 (5.0)
Cryptococcal meningitis	1 (0.5)	0 (0.0)	1 (2.5)
NTM	1 (0.5)	1 (0.6)	0 (0.0)
No. of comorbidities, *n* (%)			
1	22 (10.7)	10 (6.1)	12 (30.0)
2	5 (2.4)	2 (1.2)	3 (7.5)
≥3	1 (0.5)	0 (0.0)	1 (2.5)
Laboratory test, median (IQR)			
WBC, ×10^9^/L	6.0 (5.1, 7.2)	6.3 (5.2, 7.5)	5.4 (4.6, 6.0)
HGB, g/L	155.0 (146.0, 161.0)	156.0 (147.0, 161.0)	152.0 (140.2, 158.0)
PLT, ×10^9^/L	230.0 (196.0, 265.0)	239.0 (202.0, 271.0)	211.0 (187.8, 234.2)
ALT, U/L	28.0 (20.0, 45.0)	30.0 (20.0, 50.0)	24.5 (18.8, 32.5)
AST, U/L	25.0 (20.0, 34.0)	25.0 (21.0, 35.0)	23.0 (20.0, 31.0)
TBIL, μmol/L	10.4 (8.4, 13.9)	10.2 (8.2, 12.8)	12.0 (9.9, 16.9)
CRE, μmol/L	69.0 (62.0, 78.0)	69.0 (62.0, 78.0)	69.5 (60.5, 79.0)
eGFR, mL/min/1.73m^2^	115.4 (107.7, 121.8)	114.6 (107.5, 121.2)	117.0 (108.9, 124.2)
Random blood glucose, mmol/L	5.3 (4.9, 5.7)	5.3 (5.0, 5.7)	5.4 (4.9, 6.1)
TG, mmol/L	1.6 (1.0, 2.5)	1.7 (1.1, 2.5)	1.1 (0.7, 2.2)
Total cholesterol, mmol/L	4.5 (3.9, 5.2)	4.6 (4.0, 5.3)	4.1 (3.7, 4.5)
HDL-C, mmol/L	1.1 (0.9, 1.3)	1.1 (0.9, 1.3)	0.9 (0.9, 1.2)
LDL-C, mmol/L	2.7 (2.3, 3.4)	2.8 (2.3, 3.5)	2.5 (2.2, 3.0)
Treatment situation			
Baseline CD4 counts, cells/μL, median (IQR)	638.0 (468.0, 878.0)	719.0 (559.0, 907.0)	350.0 (264.0, 465.0)
<200 cells/μL, *n* (%)	11 (5.4)	4 (2.4)	7 (17.5)
≥200 cells/μL, *n* (%)	194 (94.6)	161 (97.6)	33 (82.5)
HIV-1 RNA, log_10_ copies/mL, median (IQR)	5.1 (3.6, 4.6)	2.5 (1.9, 4.4)	4.1 (3.7, 4.6)
<100,000 copies/mL, *n* (%)	198 (96.6)	165 (100.0)	33 (82.5)
≥100,000 copies/mL, *n* (%)	7 (3.4)	0 (0.0)	7 (17.5)
<50 copies/mL HIV-1 RNA, *n* (%)	159 (77.6)	159 (96.4)	0 (0.0)
Undetectable HIV-1 RNA, *n* (%)	143 (69.8)	143 (86.7)	0 (0.0)
DOR usage time, days, median (IQR)	149.0 (90.0, 202.0)	145.0 (90.0, 200.5)	160.5 (91.3, 225.8)
ART usage time, years, median (IQR)	6.7 (4.2, 8.8)	6.7 (4.2, 8.8)	–

Data are presented as median [interquartile range, (IQR)] or cases (percentage). IQR, interquartile range; HBV, Hepatitis B Virus; HCV, Hepatitis C Virus; NTM, non-tuberculous mycobacterium; WBC, white blood cell; HGB, hemoglobin; PLT, platelet; ALT, alanine aminotransferase; AST, aspartate aminotransferase; TBIL, total bilirubin; CRE, serum creatinine; eGFR, estimated glomerular filtration rate; TG, triglycerides; TC, total cholesterol; HDL-C, high-density lipoprotein cholesterol; LDL-C, low-density lipoprotein cholesterol; HIV-1 RNA, human immunodeficiency virus-1 ribonucleic acid. DOR, doravirine; ART, antiretroviral therapy.

The majority of the patients were male (197/205, 96.1%), with a median age of 36.0 (31.0, 41.0) years. Among them, the proportion of elderly patients was 12.7%. Co-infections included syphilis (*n* = 20), hepatitis B virus (HBV, *n* = 5), hepatitis C virus (HCV, *n* = 1), and genital warts (*n* = 3). Common comorbidities were fatty liver disease (*n* = 28), hypertension (*n* = 4), and diabetes (*n* = 3). Five patients had two comorbidities and 1 had three (Table 1).

**Figure 2:**
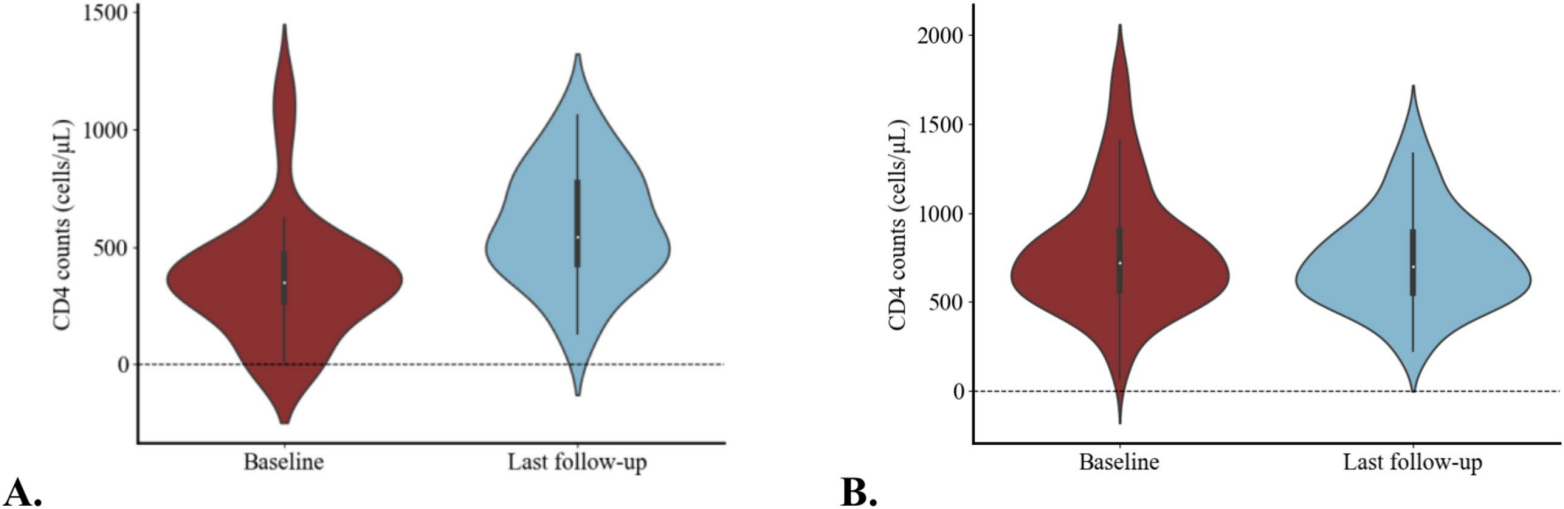
Changes in CD4 counts at baseline and last follow-up in the treatment-naïve patients **(A)** and treatment-experienced patients **(B)**.

**Figure 3:**
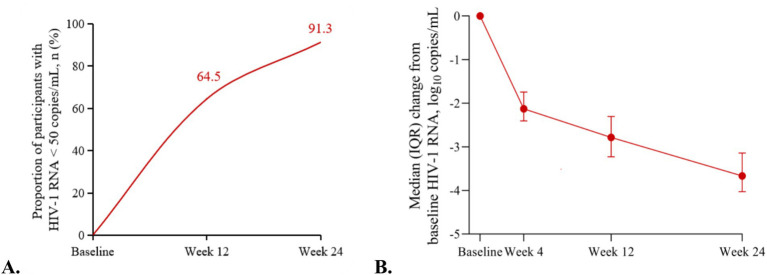
Virological outcomes in the treatment-naïve patients. **(A)** Proportion of participants with HIV-1 RNA < 50 copies/mL at weeks 12 and 24. **(B)** Change from baseline in HIV-1 RNA (log_10_ copies/mL) by visit.

Among the 40 treatment-naïve patients, CD4 counts ranged from 2 to 1,197 cells/μL, the median CD4 counts was 350.0 (264.0, 465.0) cells/μL, and 17.5% of them had CD4 counts < 200 cells/μL. The median HIV-1 RNA level was 4.1 (3.7, 4.6) log_10_ copies/mL, with 17.5% had HIV-1 RNA ≥ 10^5^ copies/mL. The median treatment duration with DOR/3TC/TDF was 160.5 (91.3, 225.8) days (Table 1). During the treatment period, rapid immunological responses were observed, and by the last follow-up, the median CD4 counts increased to 541.0 (415.8, 789.5) cells/μL (*p* < 0.05) (Figure 2A). Virological responses were favorable, with HIV-1 RNA decreased by 2.1 (1.7, 2.3) log_10_ copies/mL after 4 weeks. At weeks 12 and 24, 20/31 (64.5, 95% CI: 45.4, 80.8%) and 21/23 (91.3, 95% CI: 72.0, 98.9%) of participants achieved HIV-1 RNA < 50copies/mL, respectively (Figures 3A,B).

**Figure 4:**
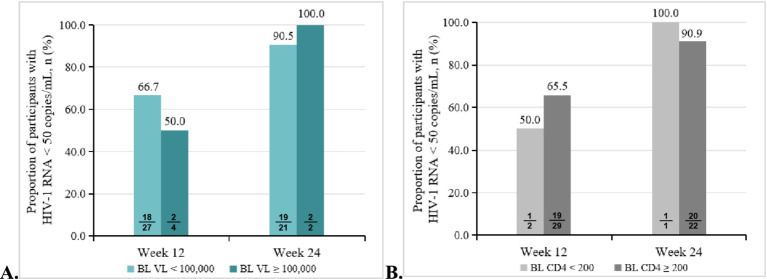
Subgroup analysis of virological outcomes in the treatment-naïve patients. **(A)** Proportion of participants with HIV-1 RNA < 50 copies/mL at weeks 12 and 24 at different baseline viral loads. **(B)** Proportion of participants with HIV-1 RNA < 50 copies/mL at weeks 12 and 24 at different baseline CD4 counts.

Subgroup analyses were performed based on baseline HIV-1 RNA levels and CD4 counts. At 12 weeks, the virological suppression rate was 2/4 (50.0, 95% CI: 6.7, 93.3%) among participants with baseline HIV-1 RNA ≥ 10^5^ copies/mL, compared to 18/27 (66.7, 95% CI: 45.7, 83.6%) in those with baseline HIV-1 RNA < 10^5^ copies/mL. By 24 weeks, the virological suppression rate was 2/2 (100.0, 95% CI: 15.8, 100%) in the baseline HIV-1 RNA ≥ 10^5^ copies/mL subgroup and 19/21 (90.5, 95% CI: 69.9, 98.9%) in the baseline HIV-1 RNA < 10^5^ copies/mL subgroup. Similarly, when stratified by baseline CD4 counts, the virological suppression rate at 12 weeks was 1/2 (50.0, 95% CI: 0.6, 79.4%) in participants with CD4 counts <200 cells/μL and 19/29 (65.5, 95% CI: 45.4, 81.5%) in those with CD4 counts ≥200 cells/μL. At 24 weeks, the suppression rate was 1/1 (100.0, 95% CI: 2.5, 100%) in the CD4 counts < 200 cells/μL subgroup and 20/22 (90.9, 95% CI: 70.8, 99.3%) in the CD4 counts ≥200 cells/μL subgroup (Figures 4A,B).

**Figure 5:**
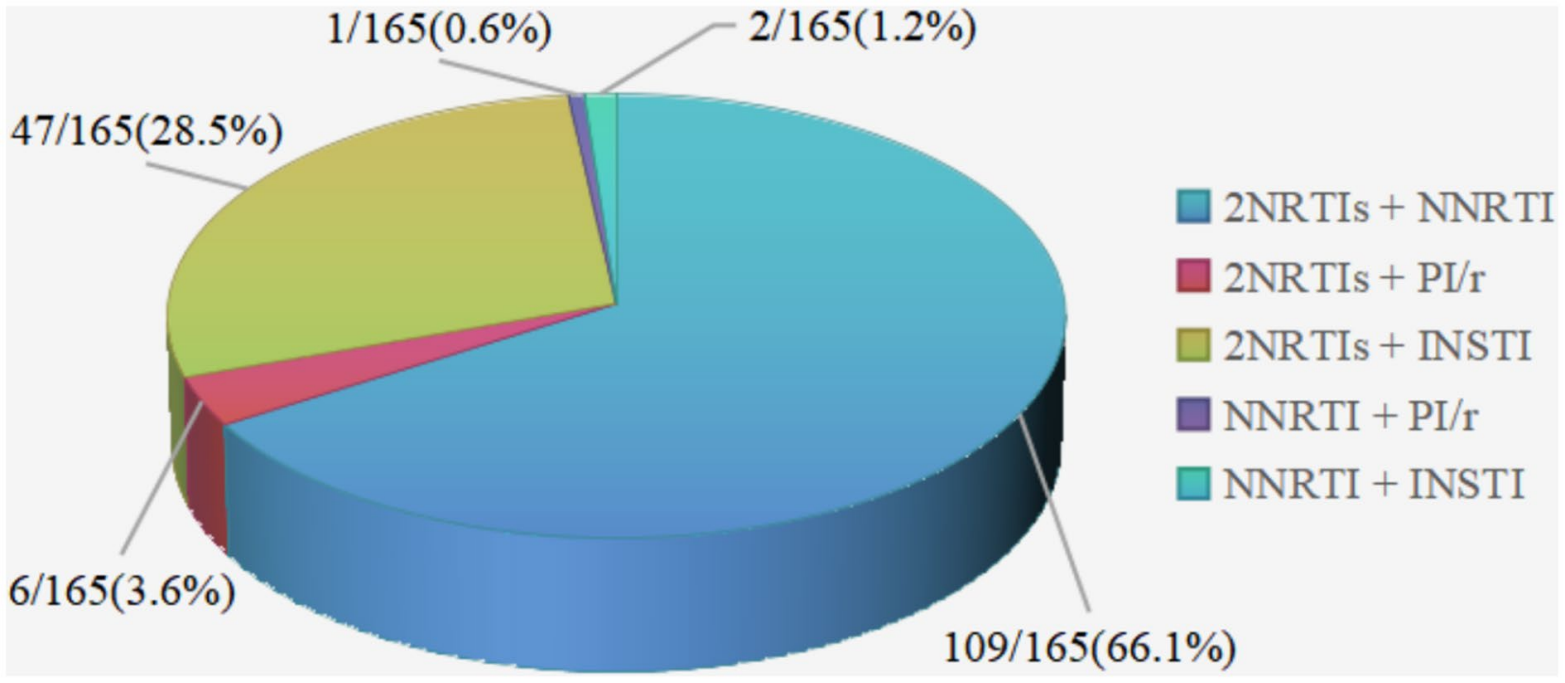
Distribution of ART regimens before the switch to DOR/3TC/TDF in treatment-experienced patients. NRTI, nucleotide reverse transcriptase inhibitor; NNRTI, non-nucleoside reverse transcriptase inhibitor; PI, protease inhibitor; /r, pharmacologically boosted with ritonavir; INSTI, integrase inhibitor.

**Figure 6:**
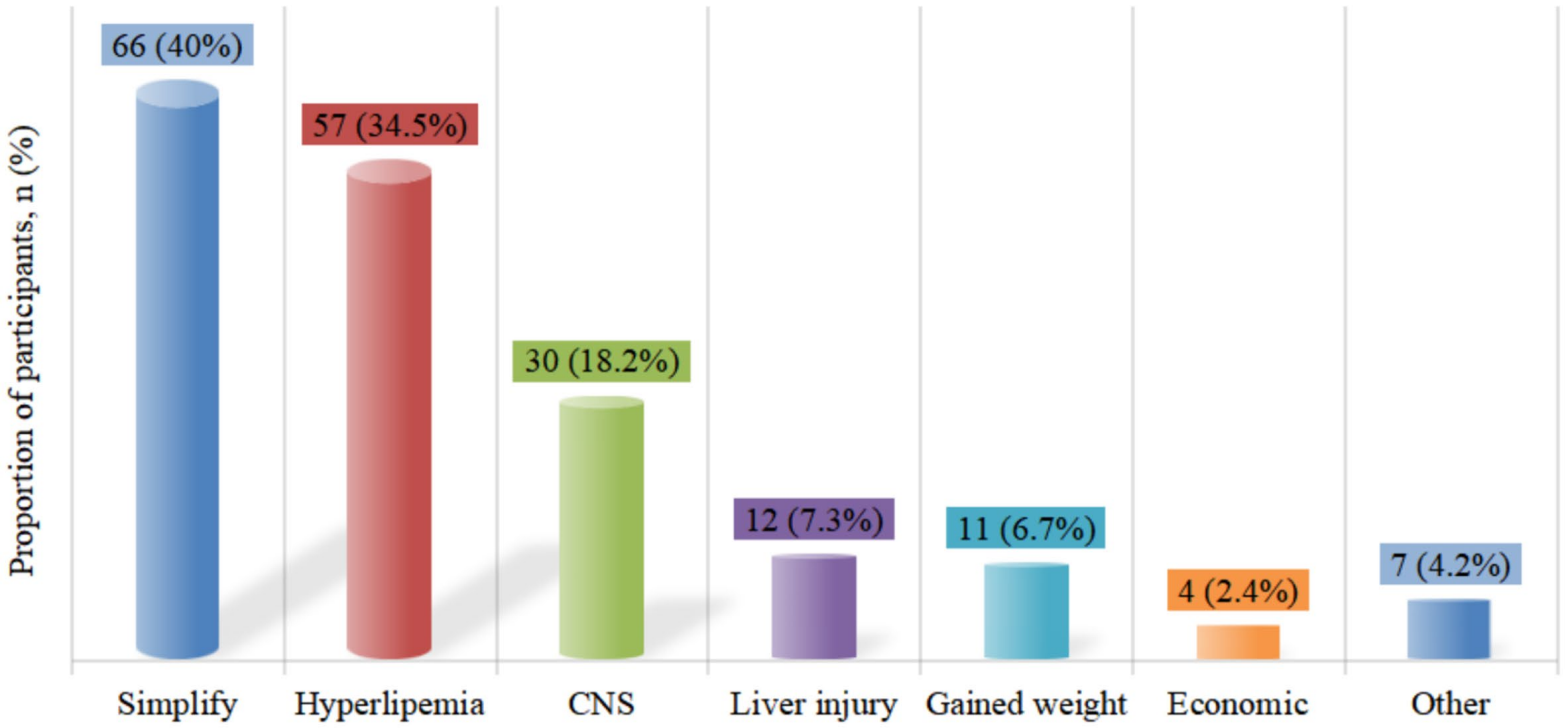
Reasons for switching to DOR/3TC/TDF in treatment-experienced patients. CNS, central nervous system.

A total of 165 patients (80.5%) had treatment-experienced, with a median ART time of 6.7 (4.2, 8.8) years before the switch (Table 1). The most common previous regimen was 2 nucleotide reverse transcriptase inhibitor (NRTIs) + NNRTI (66.1%, *n* = 109), followed by 2NRTIs + INSTI (28.5%, *n* = 47) (Figure 5). Treatment simplification was the most frequent reason for the switch (40%), followed by hyperlipemia (34.5%) and CNS symptoms (18.2%) (Figure 6).

In treatment-experienced patients, the median CD4 counts was 719.0 (559.0, 907.0) cells/μL at the time of the switch, and it remained stable during follow-up (*p* > 0.05) (Figure 2B). Among the 165 patients, 159 (96.4, 95% CI: 92.2, 98.7%) had HIV-1 RNA undetectable or <50 copies/mL at the switch, which means 6 patients had HIV-1 RNA ≥ 50 copies/mL. Among them, 4 patients had HIV-1 RNA > 200 copies/mL, two cases were due to poor adherence, and 1 patient who had only received ART for 10 days, switched the treatment regimen due to drug-induced rash. And another patient, because of liver dysfunction and hyperlipidemia, switched to DOR/3TC/TDF after receiving ART for 1 month. The HIV-1 RNA of the other two patients was between 50 and 200 copies/mL, and both of them received treatment for less than 3 months. After the switch, a similarly high proportion of patients (161/165, [97.6, 95% CI: 93.7, 99.3%]) achieved HIV-1 RNA undetectable or < 50 copies/mL (*p* > 0.05), and virological non-suppression was not attributed to efficacy failure.

By the data analysis deadline, patients had used DOR/3TC/TDF for a median of 149.0 (90.0, 202.0) days. No significant changes in liver enzymes or renal function were observed (*p* > 0.05), while body weight, random blood glucose and blood lipid levels [including total cholesterol (TG) or triglycerides (TC), low-density lipoprotein cholesterol (LDL-C)] significantly decreased (*p* < 0.05) (Table 2). Among the 30 patients who switched due to CNS symptoms (4 on INSTIs-based regimen, and 26 on NNRTIs-based regimen), their PSQI, HADS-A, and HADS-D scores, as well as the proportion of patients with these scores greater than 7 points, have significantly decreased after the switch (*p* < 0.05) (Table 3).

**Table 2:** Changes in laboratory values from baseline to the last follow-up at data cut-off for all patients.

Variables	Baseline		Current		Change	Statistical analysis	
	*N*	Median (IQR)	*N*	Median (IQR)	Median (IQR)	*Z*	*p*-value
Weight, Kg	205	70.0 (63.5, 80.0)	167	70.0 (64.0, 80.0)	0.00 (−1.00, 0.00)	−4.801^b^	<0.001
WBC, ×10^9^/L	205	6.0 (5.1, 7.2)	159	6.3 (5.4, 7.7)	0.30 (−0.37, 1.06)	−3.440^a^	0.001
HGB, g/L	205	155.0 (146.0, 161.0)	159	155.0 (149.0, 164.0)	2.00 (−3.00, 7.00)	−3.292^a^	0.001
PLT, ×10^9^/L	205	230.0 (196.0, 265.0)	159	237.0 (201.0, 271.0)	5.00 (−13.00, 22.00)	−2.377^a^	0.017
ALT, U/L	205	28.0 (20.0, 45.0)	160	29.0 (22.0, 45.0)	1.00 (−6.00, 9.00)	−1.052^a^	0.293
AST, U/L	205	25.0 (20.0, 34.0)	160	25.5 (21.0, 34.0)	0.00 (−5.00, 4.00)	−0.726^b^	0.468
TBIL, μmol/L	205	10.4 (8.4, 13.9)	160	11.8 (8.7, 14.3)	0.60 (−2.70, 3.80)	−1.432^a^	0.152
CRE, μmol/L	205	69.0 (62.0, 78.0)	160	68.5 (61.0, 75.8)	−2.00 (−6.75, 3.00)	−2.264^b^	0.024
eGFR, mL/min/1.73m^2^	205	115.4 (107.7, 121.8)	160	116.0 (106.4, 122.2)	1.25 (−2.82, 4.63)	−1.822^a^	0.069
Random blood glucose, mmol/L	203	5.3 (4.9, 5.7)	157	5.1 (4.7, 5.5)	−0.32 (−0.69, 0.12)	−5.069^b^	<0.001
TG, mmol/L	204	1.6 (1.0, 2.5)	160	1.3 (0.9, 1.9)	−0.13 (−0.75, 0.20)	−3.687^b^	<0.001
TC, mmol/L	204	4.5 (3.9, 5.2)	160	4.0 (3.6, 4.6)	−0.41 (−0.72, −0.03)	−7.535^b^	<0.001
HDL-C, mmol/L	204	1.1 (0.9, 1.3)	160	0.9 (0.8, 1.0)	−0.25 (−0.38, −0.06)	−9.051^b^	<0.001
LDL-C, mmol/L	204	2.7 (2.3, 3.4)	160	2.6 (2.1, 3.1)	−0.22 (−0.53, 0.09)	−5.091^b^	<0.001

IQR, interquartile range; WBC, white blood cell; HGB, hemoglobin; PLT, platelet; ALT, alanine aminotransferase; AST, aspartate aminotransferase; TBIL, total bilirubin; CER, serum creatinine; eGFR, estimated glomerular filtration rate; TG, triglycerides; TC, total cholesterol; HDL-C, high-density lipoprotein cholesterol; LDL-C, low-density lipoprotein cholesterol.

a *Z* value was calculated based on positive rank.

b *Z* value was calculated based on negative rank.

**Table 3:** Comparison of PSQI, HADS-A, and HADS-D scores and the proportion of those above 7 points between baseline and the last follow-up after switching to DOR due to CNS symptoms.

Variables	Baseline (*n* = 30)	Last visit (*n* = 30)	*t/χ^2^*	*p*-value
PSQI scores, mean ± SD	9.1 ± 4.1	6.7 ± 3.3	4.033	<0.001
>7 points, *n* (%)	19 (63.3)	9 (30.0)	8.100	0.002
HADS-A scores, mean ± SD	7.7 ± 4.5	5.2 ± 3.2	4.318	<0.001
>7 points, *n* (%)	15 (50.0)	7 (23.3)	6.125	0.008
HADS-D scores, mean ± SD	8.2 ± 4.8	5.8 ± 4.8	3.556	0.001
>7 points, *n* (%)	18 (60.0)	9 (30.0)	7.111	0.004

Data are presented as mean ± standard deviation (SD) or cases (percentage). CNS, central nervous system; PSQI, Pittsburgh Sleep Quality Index; HADS-A, Hospital Anxiety and Depression Scale-Anxiety; HADS-D, Hospital Anxiety and Depression Scale-Depression.

During the available follow-up period, no significant adverse events such as liver and kidney injury, abnormal lipid metabolism or significant increase in blood sugar, immune reconstitution inflammatory syndrome (IRIS), or abnormal bone metabolism were reported. Tolerance was good overall, with only 3 patients changed regimens due to adverse reactions, including difficulty swallowing (*n* = 1), insomnia (*n* = 1), and weight gain (*n* = 1).

## Discussion

This short-term real-world retrospective study demonstrated that the single-tablet regimen DOR/3TC/TDF manifested remarkable efficacy in suppressing of HIV-1 replication and elevating CD4 counts among treatment-naïve patients. Moreover, it effectively maintained virological suppression in treatment-experienced patients who switched to this regimen.

Among treatment-naïve patients, based on available data, the proportion of participants with HIV-1 RNA < 50 copies/mL was 21/23 (91.3, 95% CI: 72.0, 98.9%) at week 24, consistent with the results of two phase 3 clinical trials (DRIVE-FORWARD and DRIVE-AHEAD) of DOR (9–12). Participants who completed 96 weeks of double-blind treatment in these two studies were offered an additional 96 weeks of treatment with the DOR-based regimen in an open-label extension. Final analyses from these studies showed that the sustained efficacy lasted through to 192 weeks for first-line therapy, extending the previously found results at weeks 48 and 96 (13). DOR had demonstrated good virological suppression even in patients with high baseline HIV-1 RNA levels, as these Phase 3 trials (DRIVE-FORWARD and DRIVE-AHEAD) did not impose HIV-1 viral load (VL) restrictions as part of the inclusion criteria (13). The subgroup analysis of this study also showed that high VL and low CD4 counts did not affect the virological suppression rates (*p* > 0.05).

Among patients with treatment-experienced, switched to the DOR/3TC/TDF regimen also effectively maintained virological suppression. The results were consistent with previous studies (DRIVE-SHIFT), confirming that switching to DOR/3TC/TDF could maintain virological suppression in the long term (7). Similarly, a retrospective study from Italy demonstrated the effectiveness of the DOR/3TC/TDF regimen in maintaining virological suppression among people living with HIV (PLWH) on ART, with a low 1-year cumulative risk of treatment failure (14). Moreover, real-world studies also showed that among HIV-infected individuals who had no previous treatment failure, no confirmed virologic failure was observed at 96 weeks after switching to DOR (15).

The recovery of CD4 counts in treatment-naive patients was beyond doubt (9–12). However, without a control group, superiority or inferiority of the improvement in CD4 counts could not be evaluated. For treatment-experienced patients, the CD4 counts showed a minimal response to the treatment and remained stable after the switch. This might be related to the fact that the study population had been taking stable antiretroviral drugs for an average of more than 5 years and was on the basis of virological suppression, which was consistent with the results of the phase III clinical study of ANV/3TC/TDF (16). However, a study from Italy found that, in addition to maintaining viral suppression, there was an immunological improvement of approximately 50 CD4 cells per mL during the one-year follow-up after the switch (14). Although the magnitude of the improvement was small, it was statistically significant. Of course, this needs to be confirmed in more future studies.

The main reasons for switching to the DOR-based regimen were, apart from simplifying the regimen and improving compliance, to address the issues of improving metabolic complications and the central nervous system. This indicated that the DOR-based regimen had been widely accepted by patients and clinicians. In this study, we found that both treatment-naive and treatment-experienced patients had significantly reduced body weight, random blood glucose levels, and lipid profiles during the follow-up period. As had been previously reported, the DOR-based regimen resulted in minimal changes in fasting lipids during the first 96 weeks of treatment in the DRIVE-FORWARD and DRIVE-AHEAD trials. In contrast, participants on ritonavir-boosted darunavir (DRV/r) and efavirenz (EFV) -based regimens experienced increased fasting lipid at week 96 (10, 12). During the 192-week extension studies, lipid parameters changed minimally for those continued DOR-based regimen, while switching to DOR resulted in significant reductions in LDL-C, non-high-density lipoprotein cholesterol (n-HDL), TG and TC (13). A multicenter Italian study examined the real-world use of DOR in treatment-experienced PLWH and found significant improvements in lipid profiles. In a 24-week follow-up, 52 patients showed significant reductions in cholesterol (*p* = 0.008) and triglycerides (*p* = 0.01) after switching to DOR-based regimens (17). These results aligned with the findings from both clinical trials and real-world setting. Dyslipidemia was common among PLWH and was considered correlated with ART, especially as patients aged (18, 19). Dyslipidemia could increase the risk of cardiovascular disease in HIV-1 infected individuals, so in terms of metabolic safety, switching to DOR showed metabolic neutrality or even improvement compared to traditional NNRTIs (20, 21). This metabolic advantage might be related to its indirect regulatory effect on enzymes related to lipid metabolism (such as HMG-CoA reductase). Meanwhile, *in vitro* experiments had shown that its toxicity to pancreatic islet *β* cells was significantly lower than that of EFV and rilpivirine (RPV), which might be associated with reducing endoplasmic reticulum stress and oxidative stress (22, 23). These findings highlighted the benefits of DOR in improving lipid profile, whether used as first-line therapy or for regimen switches.

Weight gain was considered a sign of “restoring health” and has been recognized as a potential risk factor for hyperglycemia, hypertension, metabolic syndrome, and cardiovascular incidence (24, 25). Therefore, weight management cannot be ignored. In this study, the numerical changes were not significant after initial or switch treatment (with a greater contribution from the switch group), but the difference was statistically significant, showing a favorable trend of change. Although the follow-up time was relatively short, the metabolic advantage of the DOR regimen could already be observed. Previous clinical trials had shown similar weight changes across all groups (DOR, DRV, and EFV) at week 96 (10, 12). Compared to greater weight gain (mean > 4 kg at week 96) in regimens containing DTG, BIC, or tenofovir alafenamide as initial therapy (4), minimal weight gain (approximately 2 kg) was observed in the DRIVE-FORWARD and DRIVE-AHEAD cumulative groups at week 192 (13). The DRIVE-SHIFT study also reported no significant weight gain over 144 weeks (7). A retrospective study also evaluated the changes in body weight of HIV infected individuals after switching from the INSTI regimen to the DOR regimen. Within 1 year after ART switch, the body weight loss decreased by 2.6% (95% CI: −5.1, −0.1%, *p* = 0.041) (26). In the phase III study of ANV/3TC/TAF, at week 48, participants taking ANV/3TC/TAF had less weight gain compared to those taking EVG/C/FTC/TAF (1.16 kg vs. 2.05 kg) (16), which all demonstrated the advantage of weight management of the new generation of NNRTIs. Of course, due to the short follow-up time, further investigation and evaluation of the long-term effects are still needed.

Patients with CNS symptoms experienced significant improvement in PSQI, HADS-A, and HADS-D scores after switch, consistent with the results of the DRIVE-AHEAD Phase 3 study (11). DOR/3TC/TDF showed better neuropsychiatric tolerance and lower incidence of dizziness, sleep disorders/disturbances, and altered sensorium compared to efavirenz/emtricitabine/tenofovir disoproxil fumarate (EFV/FTC/TDF).

In this group of patients, serum creatinine and eGFR remained stable, with no evidence of proteinuria or renal injury, indicated good tolerability of the DOR/3TC/TDF regimen. However, the nephrotoxicity of TDF was well-recognized and it had a cumulative effect with the duration of treatment (27). Therefore, we still needed a longer observation period. Other studies had supplemented us by evaluating in detail the renal safety of the DOR/3TC/TDF regimen (14). There were no pharmacokinetic enhancers such as ritonavir or cobicistat in the DOR/3TC/TDF combination, and DOR had a minimal impact on safety parameters. This might have reduced the common risk of nephrotoxicity of TDF and provided a safer renal profile for PLWH (28, 29).

Our study has some limitations. Firstly, this was a single center retrospective study with insufficient sample size. Furthermore, due to the short-term follow-up, we are unable to evaluate the long-term benefits of DOR for both treatment-naïve and treatment-experienced patients in real world.

## Conclusion

This retrospective analysis supported the efficacy and safety of DOR/3TC/TDF in treating HIV-1 infected individuals, and provided valuable insights into the real-world outcomes expected in clinical practice.

## Data Availability

The original contributions presented in the study are included in the article/supplementary material, further inquiries can be directed to the corresponding author.
